# Investigating Relationships Between Genetic Risk, Childhood Maltreatment, and Eating Disorders in Women

**DOI:** 10.1016/j.bpsgos.2026.100761

**Published:** 2026-05-25

**Authors:** Ludvig Daae Bjørndal, Laura Hegemann, Johanne H. Pettersen, Elizabeth C. Corfield, Laurie J. Hannigan, Zeynep Yilmaz, Kayla Costello, Eivind Ystrom, Ted Reichborn-Kjennerud, Ole A. Andreassen, Helga Ask, Alexandra Havdahl, Cynthia M. Bulik, Hunna J. Watson

**Affiliations:** a Department of Psychology, PROMENTA Research Center, University of Oslo, Oslo, Norway; b Psychiatric Genetic Epidemiology Group, Research Department, Lovisenberg Diaconal Hospital, Oslo, Norway; c PsychGen Centre for Genetic Epidemiology and Mental Health, Norwegian Institute of Public Health, Oslo, Norway; d Department of Psychology, University of Oslo, Oslo, Norway; e Centre for Precision Psychiatry, Institute of Clinical Medicine, University of Oslo, Oslo Norway; f Medical Research Council Integrative Epidemiology Unit, Population Health Sciences, Bristol Medical School, University of Bristol, Bristol, United Kingdom; g National Centre for Register-Based Research, Department of Public Health, Faculty of Health, Aarhus University, Aarhus, Denmark; h Department of Biomedicine, Aarhus University, Aarhus, Denmark; i Department of Medical Epidemiology and Biostatistics, Karolinska Institutet, Stockholm, Sweden; j Department of Psychiatry, University of North Carolina at Chapel Hill, Chapel Hill, North Carolina; k Department of Psychology, University at Albany, State University of New York, Albany, New York; l Department of Child Health and Development, Norwegian Institute of Public Health, Oslo, Norway; m Division of Mental Health and Addiction, Oslo University Hospital, Oslo, Norway; n Institute of Clinical Medicine, University of Oslo, Oslo, Norway; o Department of Nutrition, Gillings School of Global Public Health, University of North Carolina at Chapel Hill, Chapel Hill, North Carolina; p School of Population Health, Curtin University, Perth, Western Australia, Australia; q Division of Pediatrics, School of Medicine, The University of Western Australia, Perth, Western Australia, Australia

**Keywords:** Childhood maltreatment, Eating disorder risk, Gene-by-environment interaction, MoBa, Norwegian Mother, Father and Child Cohort Study, Polygenic score

## Abstract

**Background:**

Genetic and environmental factors contribute to eating disorder risk, yet their interplay is poorly understood. We examined whether childhood maltreatment and polygenic scores for anorexia nervosa (PGS-AN) and binge-eating broad (PGS-BEB) are associated with eating disorders. We also examined the interactions between childhood maltreatment and PGSs in predicting eating disorders.

**Methods:**

This nested case-control study used data from up to 63,989 mothers in the MoBa (Norwegian Mother, Father and Child Cohort Study). Mothers reported on their own experiences of 4 childhood maltreatment types: long-term humiliation or degradation, threats to self or someone close, physical abuse, and sexual abuse. Diagnoses of AN, bulimia nervosa (BN), binge-eating disorder (BED), purging disorder (PD), and binge-eating spectrum disorders (BESP) were obtained using self-report data from 5 time points and population health registers.

**Results:**

The prevalence of AN, BN, BED, PD, and BESP was 2.19%, 4.15%, 10.39%, 0.60%, and 12.96%, respectively. All childhood maltreatment types were strongly associated with elevated eating disorder likelihood (with odds ratios [ORs] ranging from 1.71 to 3.29) as were eating disorder PGSs (with ORs ranging from 1.05 to 1.31). There were no multiplicative interaction effects between childhood maltreatment and PGSs. Small additive interactions were observed between PGS-AN and PGS-BEB and degradation/humiliation for BN and BESP in exploratory analyses.

**Conclusions:**

Eating disorder PGSs and childhood maltreatment are associated with higher odds of eating disorders. Furthermore, we found tentative evidence of small additive interaction effects between polygenic liability and childhood maltreatment, suggesting that their combined influence further elevates the risk of some eating disorders.

## Plain Language Summary

Genetic and environmental factors contribute to the development of eating disorders, but how these factors interact to predict eating disorder outcomes is not well understood. We examined associations between genetic risk for eating disorders, childhood maltreatment, and eating disorders in up to 63,989 mothers in the Norwegian Mother, Father and Child Cohort Study. Both genetic risk and childhood maltreatment exposure were associated with higher odds of several eating disorders. We also observed some small additive interaction effects, indicating that the combined impact of genetic risk and childhood maltreatment exposure may further increase the likelihood of some eating disorders.

Eating disorders encompass a range of mental health conditions characterized by disordered eating patterns (1). Among midlife women, the estimated lifetime prevalence is 3.6% for anorexia nervosa (AN), 2.1% for bulimia nervosa (BN), 2.0% for binge-eating disorder (BED), and 15.3% for any eating disorder (2). Each year, eating disorders account for the loss of more than 3.3 million healthy life years worldwide (3) and are associated with elevated mortality (4,5). Efficacy of treatment is poor with only 29% recovered at 17-year follow-up (6,7). The economic burden is substantial, with example estimates including AU $84 billion in Australia (8) and US $391 billion in the United States (9) in 2018.

Given the high morbidity and mortality associated with eating disorders, considerable effort has been focused on identifying risk factors. Exposure to stressful life events is associated with lifetime risk (10), onset (11), and relapse following treatment (12) of eating disorders. Childhood maltreatment, including sexual, physical, and emotional abuse, has been linked to increased risk of all eating disorders, with odds ratios (ORs) ranging from 1.88 to 5.13 (13). If stressful events increase the risk of eating disorders, public health policies aimed at preventing or mitigating such exposures could help lower their growing burden (10,13).

Genetically informed studies have demonstrated the significant role of genetic factors in eating disorders. Heritability estimates for AN and BN from twin studies range from 48% to 74% (14). Recent genome-wide association studies (GWASs) have identified several common genetic loci associated with AN and binge-eating spectrum disorders (BESP) (15, 16, 17). Polygenic scores (PGSs), calculated from GWAS findings by summing risk alleles weighted by their effect sizes, can provide a personalized measure of genetic risk for mental disorders (18,19), including eating disorders.

A small number of studies have explored how PGSs relate to eating disorders. Higher polygenic liability is associated with increased AN likelihood (OR = 1.5 for a 1 SD increase in PGS for AN [PGS-AN]) (16). PGS-AN in the highest decile (compared with the lowest) is associated with lifetime AN risk (OR = 2.59) (17). PGS-AN predicts eating disorder risk at age 14 (20) and disordered eating behaviors (21), although associations with eating disorder severity are inconsistent (22). A PGS indexing genetic propensity to binge eating has been associated with broad binge-eating outcomes (16). Further research is warranted to clarify associations between eating disorders and PGSs, particularly for non-AN outcomes, which have been less studied. Moreover, recent GWAS findings provide an updated PGS-AN algorithm (16), which may offer greater predictive accuracy and improve the identification of genetic risk for AN.

While it is hypothesized that the risk of eating disorders is shaped by the interplay of biological (e.g., genetic vulnerability) and psychosocial (e.g., stressful events) factors, few studies have investigated these interactions (23). This hypothesis is supported by etiologic models, which emphasize contributions of both genetic vulnerability and environmental stressors to increase liability (e.g., the stress-diathesis model). To our awareness, no studies have examined whether genetic liability, as indexed by PGSs, and childhood maltreatment interact to predict eating disorders. This gap underscores the need for further research to understand the combined impact of these factors on eating disorders.

Studies have investigated interaction effects between PGSs and stressful life events for other mental disorders, such as schizophrenia (24,25) and depression (26, 27, 28). However, such interaction effects between PGSs and stressful life events have not been consistently identified (25,29). The variance explained by psychiatric PGSs remains limited (16,17,30,31). Studying gene-by-environment (G × E) interactions in eating disorders is further complicated by their relatively low prevalence compared with some other mental disorders (e.g., depression), highlighting the need for adequately powered studies.

In this study, we examined associations between childhood maltreatment, eating disorder PGSs, and eating disorders using data from the MoBa (Norwegian Mother, Father and Child Cohort Study), a large national pregnancy cohort. The focus of this study was to examine these relationships in mothers enrolled in MoBa, many of which have responded to multiple questionnaires with assessments of eating disorder symptoms. The primary objectives were to 1) assess the relationship between childhood maltreatment and eating disorders, 2) characterize associations between eating disorder PGSs and eating disorders, and 3) examine potential interactions between eating disorder PGSs and childhood maltreatment in predicting eating disorders. We hypothesized that childhood maltreatment and higher genetic liability would independently be associated with elevated likelihood of an eating disorder and that their interaction would further amplify this risk.

## Methods and Materials

### Sample and Study Design

The current study is a nested case-control study designed within MoBa and is reported according to Strengthening the Reporting of Observational Studies in Epidemiology (STROBE) guidelines (32). MoBa is a population-based pregnancy cohort study conducted by the Norwegian Institute of Public Health (33). Participants were recruited from all over Norway from 1999 to 2008. Invitations to participate were sent to 277,702 women, and 41% of the invited individuals consented to participate. The MoBa cohort includes approximately 114,500 children, 95,200 mothers, and 75,200 fathers. Sample size decreased across data collection waves due to attrition. Blood samples were obtained from both parents during pregnancy and from mothers and children (umbilical cord) at birth (34). The establishment of MoBa and initial data collection was based on a license from the Norwegian Data Protection Agency and approval from The Regional Committees for Medical and Health Research Ethics. The MoBa cohort is currently regulated by the Norwegian Health Registry Act. The current study was approved by The Regional Committees for Medical and Health Research Ethics (2016/1702).

The MoBa cohort can be linked to national registries in Norway (35). We used data from the Norwegian Control and Payment of Health Reimbursements Database (KUHR), which covers data from primary health care services (2008–2021), and the Norwegian Patient Registry (NPR), which covers data from specialist health care services (2008–2023), to access further data on history of eating disorder diagnosis.

Genotype data were available for approximately 80% of the MoBa cohort. We used data from a genotype quality control pipeline, which restricted the sample to participants with European ancestry genotype data (36). We used the R package phenotools (version 0.3.0) for preparing MoBa data (37).

We included mothers participating in MoBa in the current study. Mothers with >1 measure (*n* = 16,476) were assigned an eating disorder if they met diagnostic criteria during any time point, with data subsequently linked to a single child selected randomly to maintain independence of observations. Inclusion criteria for the current study were nonmissing eating disorder status for at least 1 time point (*n* = 84,196), genotype data available for calculating PGSs (*n* = 69,494), and nonmissing self-reported data on own experiences of childhood maltreatment for at least 1 maltreatment type (*n* = 64,353). Exclusion criteria included nonvalid values for weight (<30 kg [67 lb] or >300 kg [661 lb]) or height (<100 cm [3.3 feet]; *n* = 364). This resulted in a final analytic sample size of 63,989 participants. A flow chart for selection of participants is presented in Figure S1.

Sociodemographic characteristics at baseline (week 15 of pregnancy) in the analytic sample are reported in Table 1 (baseline characteristics for the full MoBa sample of mothers are reported in Table S2).

**Table 1:** Sample Sociodemographic Characteristics at Week 15 of Pregnancy

Characteristic	*n* (%) or Mean (SD)
Age, Years^a^	30.28 (4.57)
Marital Status^b^	
Married	29,778 (46.54%)
Divorced or separated	159 (0.25%)
Cohabiting	31,617 (49.41%)
Single	1189 (1.86%)
Widowed and other	611 (0.95%)
Education Level, Completed^c^	
No higher education	21,404 (33.45%)
Higher education	39,025 (60.99%)
Household Income^d^	
No income	1218 (1.90%)
<150,000 NOK	9358 (14.62%)
151,000–299,999 NOK	28,122 (43.95%)
300,000–499,999 NOK	20,083 (31.39%)
>500,000 NOK	2831 (4.42%)

These numbers were based on all participants with at least 1 available eating disorder assessment, genotype data, and at least 1 available assessment of childhood maltreatment and who were not excluded (*n* = 63,989). Percentages do not add up to 100 because of missing data for individual variables.

NOK, Norwegian Krone.

a Missing data for this variable was *n* = 57.

b Missing data for this variable was *n* = 526 (109 participants additionally selected more than 1 response alternative).

c Missing data for this variable was *n* = 3560.

d Missing data for this variable was *n* = 2377.

### Measures

#### Eating Disorder Classification

We assessed lifetime eating disorder status using 2 sources: survey-based diagnostic algorithms from MoBa questionnaire items, which aligned with DSM-5 criteria (1) and diagnostic codes from NPR or KUHR. The survey-based algorithms captured AN, BN, BED, and other specified feeding or eating disorders—purging disorder (PD), and along with earlier DSM-IV algorithms, have been used extensively in previous MoBa studies (38, 39, 40). Items assessing eating disorders are included in 5 MoBa questionnaires: week 15 of pregnancy and years 1.5, 3, 8, and 14 after giving birth. Current eating disorders were assessed at each measurement occasion. Additionally, the pregnancy questionnaire assessed eating disorders in the 6 months prior to pregnancy; the 1.5-year questionnaire assessed lifetime BN, BED, and PD; and the 8-year questionnaire assessed lifetime AN. Using NPR or KUHR data, eating disorders were classified as present if individuals had ever received a relevant diagnostic code.

Using self-report and registry data, we assessed the following eating disorder outcomes: AN, BN, BED, PD, and BESP (classified with either BN or BED). Control participants were individuals who did not meet criteria for any eating disorder at any time point in MoBa and had never received an eating disorder diagnosis in the health registries. All eating disorder outcomes (including relevant diagnostic codes) are reported in Table S3.

#### Childhood Maltreatment

Experiences of retrospectively assessed childhood maltreatment (before age 18 years) in MoBa mothers were assessed at week 30 of pregnancy. Four types of maltreatment were assessed as binary variables (yes/no): long-term humiliation or degradation, having been threatened or someone close has been threatened, having experienced physical abuse, and experiences of sexual abuse. The items were adapted from the NorVold Abuse Questionnaire, which has been shown to have good reliability and validity with an interview as the gold standard (41). We examined consistency in reporting of childhood maltreatment for mothers who participated twice in MoBa. Among mothers who endorsed a given maltreatment item at either wave, the proportion of those who endorsed it at both waves ranged from 36% (threatened) to 62% (sexual abuse).

#### Polygenic Scores

PGSs were calculated using the software LDpred2 (42). Recommended quality control steps on the summary statistics were followed using an established pipeline (42,43), which included restricting variants to an extended set of HAPMAP3+ variants (1.4 million single nucleotide polymorphisms). Precomputed linkage disequilibrium (LD) matrices from the UK Biobank were used as the reference LD panel (44). Scores were calculated using the option LDPred2-auto. PGSs were regressed on the first 20 genomic principal components, genotype, and imputation batch, and standardized (based on the study sample) to have a mean of 0 and SD of 1.

PGSs for AN (PGS-AN) and for binge-eating broad (PGS-BEB) were calculated based on the respective GWASs from the Psychiatric Genomics Consortium (PGC) Eating Disorder Working Group (16). To prevent overlap, the MoBa cohort was excluded from the GWAS used to derive the eating disorder PGSs through a leave-one-cohort-out strategy.

### Statistical Analyses

We investigated associations between childhood maltreatment, PGSs, and eating disorders using a series of generalized linear models with a binomial distribution and a logit link function (the model fitting strategy is detailed in Table S4). Model 0 examined the relationship between each childhood maltreatment type and each eating disorder separately. Model 1 estimated the associations between PGSs and eating disorders in 2 steps: 1) univariable models, where each PGS was tested individually as a predictor of each eating disorder, and 2) multivariable models, where both PGSs were included together to examine the associations with each eating disorder (i.e., adjusted for the other PGS). Model 2 tested the multiplicative interaction between childhood maltreatment and PGSs by including the main effects of each predictor and their interaction term (PGS × childhood maltreatment). Each of the 4 childhood maltreatment types and PGSs were included in separate models for each eating disorder. The step progression from models 0 to 2 with multivariable adjustment follows a framework previously used for modeling associations between stressful life events and psychiatric outcomes (45).

In exploratory analyses, we examined additive interaction effects by quantifying the relative excess risk due to interaction (RERI) with the following equation (46):

(1) $$RERI=e^{{\hat{\beta }}_{1}+{\hat{\beta }}_{2}+{\hat{\beta }}_{3}}-e^{{\hat{\beta }}_{1}}-e^{{\hat{\beta }}_{2}}+1\text{.}$$

The 95% CIs for RERI estimates were obtained by bootstrapping (*n* = 1000). This approach aligns with recent studies assessing G × E interaction effects with psychiatric PGSs and outcomes (24,28). Finally, model 3 tested associations between PGS quartiles and eating disorders to assess these relationships across genetic risk strata. Presenting results by quartiles aligns with how risk is often conceptualized in clinical and public health contexts (e.g., low, moderate, and high genetic liability), thereby helping readers to contextualize differences in eating disorder risk across the PGS continuum. As a sensitivity analysis, we also examined associations between childhood maltreatment, PGSs, and registry eating disorder diagnoses (Table S8).

All main models were adjusted for education, household income, and marital status to statistically control for the potential influence of these variables and is consistent with previous analyses of eating disorders in MoBa (39). To account for multiple comparisons, we controlled for false discovery rate (FDR) using the Benjamini and Yekutieli method (47). A *p*_FDR_ value < .05 was considered statistically significant (all main results are presented after correcting for multiple testing). All analyses used only available data without imputation. Analyses were conducted in R version 4.1.2 (48) and SAS 9.4 (SAS Institute, Cary, NC).

### Preregistration and Code

This study was preregistered, with deviations and corresponding justifications described in the Supplement. Code for reproducing the main results is publicly available: https://doi.org/10.17605/OSF.IO/6GWR2.

## Results

### Prevalence of Eating Disorders and Childhood Maltreatment

The prevalence of eating disorders ranged from 0.6% (PD) to 13.0% (BESP) (Table 2). Frequency of childhood maltreatment ranged from 4.3% (having been or someone close having been threatened) to 12.0% (subdued, degraded, or humiliated), with 18.4% having experienced any form of childhood maltreatment.

**Table 2:** Prevalence of Eating Disorders and Childhood Maltreatment in the Study Sample

Eating Disorder Diagnosis and Childhood Maltreatment	*n* (%)
Eating Disorder Outcome	

Anorexia Nervosa	1401 (2.19%)
Bulimia Nervosa	2656 (4.15%)
Binge-Eating Disorder	6649 (10.39%)
Purging Disorder	385 (0.60%)
Binge-Eating Spectrum	8295 (12.96%)
No Eating Disorder	54,390 (85.00%)

Childhood Maltreatment, Before 18 Years	
Subdued, Degraded, or Humiliated	7699 (12.03%)
Threatened or Threats to Someone Close	2718 (4.25%)
Physical Abuse	3429 (5.36%)
Sexual Abuse	4370 (6.83%)
One Stressful Life Event	7619 (11.91%)
Two Stressful Life Events	2373 (3.71%)
Three Stressful Life Events	1225 (1.91%)
Four Stressful Life Events	544 (0.85%)

These numbers were based on all participants with at least 1 available eating disorder assessment, genotype data, and at least 1 available assessment of childhood maltreatment and who were not excluded (*n* = 63,989).

### Associations Between Childhood Maltreatment and Eating Disorders

All childhood maltreatment types were associated with increased likelihood of all eating disorders (model 0) (Table 3). For AN, sexual abuse exhibited the strongest association (OR = 2.67, 95% CI [2.26 to 3.14]). Associations were generally of similar magnitude across all maltreatment types for BN (2.20 ≤ OR ≤ 2.26), BED (1.83 ≤ OR ≤ 1.97), and BESP (1.87 ≤ OR ≤ 2.02). PD was most strongly associated with sexual abuse (OR = 3.29, 95% CI [2.47 to 4.31]). The likelihood of all eating disorders increased when participants reported experiencing more childhood maltreatment exposures.

**Table 3:** Associations Between Childhood Maltreatment and Eating Disorders

Predictors	OR (95% CI)				
	AN	BN	BED	PD	BESP
Degradation	2.00 (1.73 to 2.30)	2.22 (2.00 to 2.45)	1.83 (1.70 to 2.00)	1.72 (1.30 to 2.24)	1.87 (1.75 to 2.00)
Threatened	2.20 (1.77 to 2.71)	2.20 (1.88 to 2.57)	1.84 (1.65 to 2.05)	1.71 (1.10 to 2.54)*∗*	1.93 (1.74 to 2.13)
Physical Abuse	2.30 (1.89 to 2.77)	2.23 (1.93 to 2.56)	1.97 (1.78 to 2.17)	2.46 (1.74 to 3.39)	2.02 (1.84 to 2.20)
Sexual Abuse	2.67 (2.26 to 3.14)	2.26 (1.98 to 2.56)	1.89 (1.72 to 2.06)	3.29 (2.47 to 4.31)	1.97 (1.82 to 2.14)
One Childhood Maltreatment Exposure	1.94 (1.67 to 2.25)	1.91 (1.71 to 2.14)	1.65 (1.53 to 1.78)	2.13 (1.62 to 2.78)	1.69 (1.57 to 1.80)
Two Childhood Maltreatment Exposures	2.39 (1.87 to 3.01)	2.60 (2.19 to 3.07)	2.29 (2.04 to 2.56)	2.79 (1.83 to 4.10)	2.29 (2.06 to 2.54)
Three Childhood Maltreatment Exposures	2.75 (2.01 to 3.68)	2.57 (2.03 to 3.21)	2.08 (1.77 to 2.44)	2.23 (1.17 to 3.85)*∗∗*	2.21 (1.91 to 2.55)
Four Childhood Maltreatment Exposures	4.90 (3.34 to 6.97)	4.40 (3.28 to 5.80)	2.89 (2.30 to 3.59)	4.03 (1.89 to 7.56)	3.19 (2.61 to 3.89)

The *p* values were adjusted for multiple testing (number of tests = 40). Confidence intervals are unadjusted.

All false discovery rate–corrected *p*s < .001 except ∗*p* = .05 and ∗∗*p* = .03.

AN, anorexia nervosa; BED, binge-eating disorder; BESP, binge-eating spectrum disorder; BN, bulimia nervosa; OR, odds ratio; PD, purging disorder.

### Associations Between PGSs and Eating Disorders

PGS-AN was associated with increased likelihood of AN, BN, PD, and BESP in unadjusted analyses (1.05 ≤ OR ≤ 1.31), and AN (OR = 1.31, 95% CI [1.23 to 1.40]) in analyses adjusting for PGS-BEB (models 1a and 1b) (Table 4). PGS-BEB was associated with all eating disorders in unadjusted analyses (1.10 ≤ OR ≤ 1.25) and BN (OR = 1.22, 95% CI [1.16 to 1.28]), BED (OR = 1.12, 95% CI [1.08 to 1.15]), PD (OR = 1.17, 95% CI [1.04 to 1.32]), and BESP (OR = 1.14, 95% CI [1.11 to 1.17]) when adjusting for PGS-AN. Higher PGS quartiles were associated with increased likelihood of all eating disorders (Table S5).

**Table 4:** Associations Between Eating Disorder PGSs and Eating Disorders

Predictor	AN		BN		BED		PD		BESP	
	OR (95% CI)	*p*	OR (95% CI)	*p*	OR (95% CI)	*p*	OR (95% CI)	*p*	OR (95% CI)	*p*
PGS-AN^a^	1.31 (1.24 to 1.39)	<.001	1.15 (1.11 to 1.20)	<.001	1.02 (0.99 to 1.04)	.885	1.24 (1.11 to 1.37)	<.001	1.05 (1.03 to 1.08)	<.001
PGS-AN^a^, Adjusted	1.31 (1.23 to 1.40)	<.001	1.05 (1.00 to 1.10)	.183	0.96 (0.94 to 0.99)	.098	1.15 (1.02 to 1.29)	.109	0.99 (0.96 to 1.02)	>.99
PGS-BEB^b^	1.14 (1.08 to 1.21)	<.001	1.25 (1.20 to 1.30)	<.001	1.10 (1.07 to 1.13)	<.001	1.25 (1.13 to 1.39)	<.001	1.14 (1.11 to 1.16)	<.001
PGS-BEB^b^, Adjusted	1.01 (0.94 to 1.07)	>.99	1.22 (1.16 to 1.28)	<.001	1.12 (1.08 to 1.15)	<.001	1.17 (1.04 to 1.32)	.048	1.14 (1.11 to 1.17)	<.001

The ORs for PGSs can be interpreted as the effects of an SD increase in the PGS. These models adjusted for all covariates (i.e., marital status, education level, household income). Adjusted PGSs further adjusted for other PGSs. The *p* values were adjusted for multiple testing (number of tests = 20). Confidence intervals are unadjusted.

AN, anorexia nervosa; BEB, binge-eating broad; BED, binge-eating disorder; BESP, binge-eating spectrum disorder; BN, bulimia nervosa; PD, purging disorder; OR, odds ratio; PGS, polygenic score.

a Range: −4.14 to 4.34.

b Range: −4.32 to 4.57.

### Interaction Effects Between PGSs and Childhood Maltreatment on a Multiplicative Scale

No interaction effects on the multiplicative scale were statistically significant (model 2) (main and interaction effects reported in Tables S6 and S7).

### Interaction Effects Between PGSs and Childhood Maltreatment on the Additive Scale

**Figure 1:**
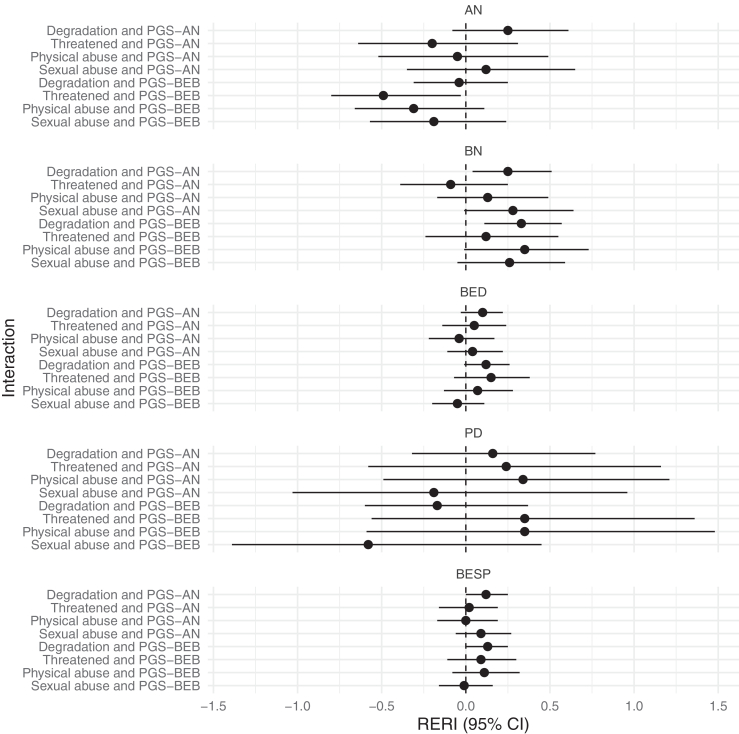
Exploratory analyses of interaction effects between childhood maltreatment and PGSs in predicting eating disorders (additive scale). The tests were not preregistered. RERI >0 indicates a positive additive interaction (combined effect exceeds the sum of individual effects), RERI <0 indicates a negative additive interaction (combined effect is less than the sum), and RERI = 0 indicates no additive interaction. Small additive interaction effects were observed between long-term degradation/humiliation and PGS-AN for BN (RERI = 0.25) and BESP (RERI = 0.12), similarly for degradation/humiliation and PGS-BEB for BN (RERI = 0.33) and BESP (RERI = 0.13), and between being threatened and PGS-BEB for AN (RERI = −0.49). AN, anorexia nervosa; BEB, binge-eating broad; BED, binge-eating disorder; BESP, binge-eating spectrum disorder; BN, bulimia nervosa; PD, purging disorder; PGS, polygenic score; RERI, relative excess risk due to interaction.

Small interaction effects were observed between long-term degradation/humiliation and PGS-AN for BN (RERI = 0.25, 95% CI [0.04 to 0.51]) and BESP (RERI = 0.12, 95% CI [0.00 to 0.25]) on the additive scale (Figure 1 and Table S9). Interaction effects on the additive scale were also observed between degradation/humiliation and PGS-BEB for BN (RERI = 0.33, 95% CI [0.11 to 0.57]) and BESP (RERI = 0.13, 95% CI [0.00 to 0.25]) and between being threatened and PGS-BEB for AN (RERI = −0.49, 95% CI [−0.80 to −0.03]).

## Discussion

Using data from a large nationwide pregnancy-based cohort, we identified substantial associations between childhood maltreatment, eating disorder PGSs, and eating disorders. While no interaction effects on the multiplicative scale were noted, we observed some interaction effects on the additive scale, indicating that the combined influence of childhood maltreatment and polygenic liability may increase the likelihood of some eating disorder outcomes, specifically BN and BESP.

Prevalence estimates ranged from 0.6% (PD) to 13.0% (BESP), with 2.19% for AN and 4.15% for BN. The observed AN prevalence is comparable to what has been reported for women in the United States (1.42%) (49). The observed prevalence of AN and BN aligns with estimates for women in ALSPAC (Avon Longitudinal Study of Parents and Children) (3.64% and 2.15%, respectively), although we observed a higher prevalence of BED (10.39% vs. 1.96%, respectively) (2). This discrepancy may partly be due to the methodology used to assess BED. Specifically, BED was assessed using self-report items that captured the presence of binge eating but did not assess the DSM-5 criterion B features (i.e., the additional features that must accompany binge episodes, such as eating until feeling uncomfortably full) or criterion C (i.e., marked distress), which could have inflated our BED prevalence estimates. Previous population-based prevalence estimates for BED using diagnostic interviews are considerably lower than that reported here (49). Overall, we observed a high prevalence of eating disorders in the MoBa cohort, which is noteworthy given the potential impact of maternal eating disorders on perinatal outcomes in children (40).

Our findings support the notion that childhood maltreatment is a considerable risk factor for eating disorders (2,10,13). Exposure to childhood maltreatment was associated with increased odds of all eating disorders (1.71 ≤ OR ≤ 3.29), which aligns with meta-analytic estimates (13). Notably, a substantial proportion (18%) reported experiencing at least one form of childhood maltreatment, most commonly degradation/humiliation (12%), followed by sexual abuse (7%).

We found that eating disorder PGSs predicted eating disorders, with the strongest evidence for AN-PGS (1.05 ≤ OR ≤ 1.31, for a 1 SD increase in polygenic liability with respect to AN, BN, PD, and BESP). Individuals in higher quartiles of polygenic risk were more likely to experience eating disorders (1.11 ≤ OR ≤ 1.94). This aligns with previous findings for AN and is indicative of the polygenic nature of eating disorders (16,17,20,50). Notably, we report associations for both AN and binge-eating PGSs based on a recent GWAS (16) with multiple non-AN eating disorders, providing a step forward in quantifying how current PGSs predict different eating disorder outcomes.

Small interaction effects between PGSs and childhood maltreatment on the additive scale were observed between PGSs and degradation/humiliation for BN and BESP. This would be consistent with a sufficient cause interaction, whereby an additive-scale synergism sees both genetic liability and degradation/humiliation jointly contribute to a causal mechanism for disorder onset in some individuals (51,52). The interaction between childhood threats and PGS-BEB for AN was negative, contrary to other additive interaction effects. The wide confidence interval and fewer individuals reporting this exposure indicated limited statistical power. However, alternative explanations may exist. Individuals with high polygenic risk for binge eating and exposure to high stress may be more likely to develop binge-eating behaviors (typical of BN or BED), rather than restrictive behaviors (typical of AN), potentially attenuating the genetic association with AN. Trends toward higher BN, BED, and BESP likelihood could support this (Figure 1).

We did not find evidence of multiplicative interaction effects between PGSs and childhood maltreatment, in partial alignment with some previous studies examining G × E effects for schizophrenia (25) and depression (28). As PGSs explain only a small proportion of phenotypic variance, further research on G × E interaction interplay in eating disorders could benefit from genetic measures that capture more variance, potentially increasing the power to detect such interactions (53).

### Clinical Implications

Identifying empirically supported risk factors is critical for efforts to reduce the incidence of eating disorders (54,55). Our findings point to both childhood maltreatment and polygenic liability as risk factors for eating disorders and provide tentative evidence of some additive interaction effects. Evidence for the predictive ability of PGS for eating disorder risk is growing but remains in its early stages (56).

### Strengths and Limitations

Our study has several strengths. We used data from a large, population-based pregnancy cohort. Eating disorder outcomes aligned with current diagnostic criteria, and we incorporated diagnoses from population health registries, covering primary and specialist healthcare services. Furthermore, as eating disorders have been assessed across multiple measurement occasions in MoBa, the assignment of diagnoses did not rely on a single time point.

However, there are limitations. The genomic pipeline in MoBa only includes participants of European ancestry (36), limiting generalizability. This choice was made based on the complexity of quality control procedures when most of the participants (95%) clustered with individuals from one 1000 Genomes reference superpopulation. Childhood maltreatment exposure was assessed retrospectively, does not capture time-varying effects of stressful life events, and may be influenced by recall bias. Retrospective and prospective measures of childhood maltreatment have shown limited agreement (57). Furthermore, we observed substantially lower consistency in reporting of childhood maltreatment for mothers who participated twice in MoBa (36%–62%) when compared with a previous study with assessment time points closer in time (41). As such, we cannot exclude the potential influence of relatively low reliability of the childhood maltreatment measures used. The observed findings should be interpreted considering this limitation. While eating disorder outcomes aligned with diagnostic criteria, diagnostic interviews were not conducted. Self-report assessments may contribute to overestimated eating disorder prevalence when compared with diagnostic interviews (49). Future research examining G × E interactions could benefit from using richer phenotyping and well-validated diagnostic measures to more comprehensively capture eating disorder presentations. As participants provided data during pregnancy or postpartum, we cannot be certain that findings generalize to all individuals in the broader population. Furthermore, the potential influence of bias due to selection (e.g., higher education level and health status among women able to achieve pregnancy) or attrition in MoBa may further limit the generalizability of the results (58, 59, 60). Finally, the small proportion of phenotypic variance accounted for by eating disorder PGSs may limit the ability to detect G × E interactions. In addition, both childhood maltreatment and genetic risk are associated with numerous mental disorders (45,61). Thus, the observed associations between these risk factors and eating disorders in this study may in part reflect associations with other mental health outcomes, which we have not assessed. Furthermore, there are many potential risk factors and pathways to eating disorders, beyond those assessed in this study. Future work could examine how shared vulnerabilities across psychiatric disorders jointly contribute to eating disorder risk. As our sample comprised mothers enrolled in MoBa, future studies could also assess the extent to which associations between PGSs, childhood maltreatment, and eating disorders are similar in men.

### Conclusions

Our findings highlight the role of childhood maltreatment and eating disorder PGSs in predicting eating disorders. We found some evidence of additive interactions between childhood maltreatment and PGSs, indicating that the combined effect of these may further elevate likelihood of BN and BESP. No multiplicative interactions between childhood maltreatment and PGSs were observed. Future research should focus on refining measures of genetic risk to capture greater phenotypic variance and further explore G × E interaction interplay in eating disorders. Such knowledge can potentially inform prevention and early intervention efforts with increased precision.

## Supplementary Material

Supplemental Methods, Figure S1 and Tables S1–S12

Key Resources Table

## References

[1] American Psychiatric Association (2013).

[2] Micali N., Martini M.G., Thomas J.J., Eddy K.T., Kothari R., Russell E. (2017). Lifetime and 12-month prevalence of eating disorders amongst women in mid-life: A population-based study of diagnoses and risk factors. BMC Med.

[3] van Hoeken D., Hoek H.W. (2020). Review of the burden of eating disorders: Mortality, disability, costs, quality of life, and family burden. Curr Opin Psychiatry.

[4] Arcelus J., Mitchell A.J., Wales J., Nielsen S. (2011). Mortality rates in patients with anorexia nervosa and other eating disorders: A meta-analysis of 36 studies. Arch Gen Psychiatry.

[5] Fichter M.M., Quadflieg N. (2016). Mortality in eating disorders - Results of a large prospective clinical longitudinal study. Int J Eat Disord.

[6] Eielsen H.P., Vrabel K., Hoffart A., Rø Ø., Rosenvinge J.H. (2021). The 17-year outcome of 62 adult patients with longstanding eating disorders-A prospective study. Int J Eat Disord.

[7] Miskovic-Wheatley J., Bryant E., Ong S.H., Vatter S., Le A., Aouad P. (2023). Eating disorder outcomes: Findings from a rapid review of over a decade of research. J Eat Disord.

[8] Hay P., Aouad P., Le A., Marks P., Maloney D., Barakat S. (2023). Epidemiology of eating disorders: Population, prevalence, disease burden and quality of life informing public policy in Australia - A rapid review. J Eat Disord.

[9] Harvard T.H., Chan School of Public Health The social and economic cost of eating disorders in the United States of America: A report for the Strategic Training Initiative for the Prevention of Eating Disorders and the Academy for Eating Disorders.. https://www.hsph.harvard.edu/striped/report-economic-costs-of-eating-disorders/.

[10] Afifi T.O., Sareen J., Fortier J., Taillieu T., Turner S., Cheung K., Henriksen C.A. (2017). Child maltreatment and eating disorders among men and women in adulthood: Results from a nationally representative United States sample. Int J Eat Disord.

[11] Treasure J., Duarte T.A., Schmidt U. (2020). Eating disorders. Lancet.

[12] Grilo C.M., Pagano M.E., Stout R.L., Markowitz J.C., Ansell E.B., Pinto A. (2012). Stressful life events predict eating disorder relapse following remission: Six-year prospective outcomes. Int J Eat Disord.

[13] Molendijk M.L., Hoek H.W., Brewerton T.D., Elzinga B.M. (2017). Childhood maltreatment and eating disorder pathology: A systematic review and dose-response meta-analysis. Psychol Med.

[14] Bulik C., Yilmaz Z., HArdaway A. (2015). Genetics and epigenetics of eating disorders. Adv Genomics Genet.

[15] Duncan L., Yilmaz Z., Gaspar H., Walters R., Goldstein J., Anttila V. (2017). Significant locus and metabolic genetic correlations revealed in genome-wide association study of anorexia nervosa. Am J Psychiatry.

[16] Termorshuizen JD, Davies HL, Lee S-H, Dennis JK, Hübel C, Johnson JS, *et al*. (in press): Genome-wide association studies of binge eating behaviour and anorexia nervosa yield insights into the unique and shared biology of eating disorder phenotypes. Nat Ment Health. 10.1038/s44220-026-00698-2 PMC13553282 42718672

[17] Watson H.J., Yilmaz Z., Thornton L.M., Hübel C., Coleman J.R.I., Gaspar H.A. (2019). Genome-wide association study identifies eight risk loci and implicates metabo-psychiatric origins for anorexia nervosa. Nat Genet.

[18] Allegrini A.G., Baldwin J.R., Barkhuizen W., Pingault J.B. (2022). Research Review: A guide to computing and implementing polygenic scores in developmental research. J Child Psychol Psychiatry.

[19] Wray N.R., Lin T., Austin J., McGrath J.J., Hickie I.B., Murray G.K., Visscher P.M. (2021). From basic science to clinical application of polygenic risk scores: A primer. JAMA Psychiatry.

[20] Yilmaz Z., Schaumberg K., Halvorsen M., Goodman E.L., Brosof L.C., Crowley J.J. (2023). Predicting eating disorder and anxiety symptoms using disorder-specific and transdiagnostic polygenic scores for anorexia nervosa and obsessive-compulsive disorder. Psychol Med.

[21] Curtis M., Colodro-Conde L., Medland S.E., Gordon S., Martin N.G., Wade T.D., Cohen-Woods S. (2024). Anorexia nervosa polygenic risk, beyond diagnoses: Relationship with adolescent disordered eating and behaviors in an Australian female twin population. Psychol Med.

[22] Johansson T., Birgegård A., Zhang R., Bergen S.E., Landén M., Petersen L.V. (2022). Polygenic association with severity and long-term outcome in eating disorder cases. Transl Psychiatry.

[23] Culbert K.M., Racine S.E., Klump K.L. (2015). Research Review: What we have learned about the causes of eating disorders - A synthesis of sociocultural, psychological, and biological research. J Child Psychol Psychiatry.

[24] Guloksuz S., Pries L.K., Delespaul P., Kenis G., Luykx J.J., Lin B.D. (2019). Examining the independent and joint effects of molecular genetic liability and environmental exposures in schizophrenia: Results from the EUGEI study. World Psychiatry.

[25] Pries L.K., van Os J., ten Have M., de Graaf R., van Dorsselaer S., Bak M. (2020). Association of recent stressful life events with mental and physical health in the context of genomic and exposomic liability for schizophrenia. JAMA Psychiatry.

[26] Lipsky R.K., Garrett M.E., Dennis M.F., Hauser M.A., Beckham J.C., Ashley-Koch A.E., Kimbrel N.A. (2023). Impact of traumatic life events and polygenic risk scores for major depression and posttraumatic stress disorder on Iraq/Afghanistan Veterans. J Psychiatr Res.

[27] Mullins N., Power R.A., Fisher H.L., Hanscombe K.B., Euesden J., Iniesta R. (2016). Polygenic interactions with environmental adversity in the aetiology of major depressive disorder. Psychol Med.

[28] Musliner K.L., Andersen K.K., Agerbo E., Albiñana C., Vilhjalmsson B.J., Rajagopal V.M. (2023). Polygenic liability, stressful life events and risk for secondary-treated depression in early life: A nationwide register-based case-cohort study. Psychol Med.

[29] Musliner K.L., Seifuddin F., Judy J.A., Pirooznia M., Goes F.S., Zandi P.P. (2015). Polygenic risk, stressful life events and depressive symptoms in older adults: A polygenic score analysis. Psychol Med.

[30] Mullins N., Bigdeli T.B., Børglum A.D., Coleman J.R.I., Demontis D., Mehta D. (2019). GWAS of suicide attempt in psychiatric disorders and association with major depression polygenic risk scores. Am J Psychiatry.

[31] Ni G., Zeng J., Revez J.A., Wang Y., Zheng Z., Ge T. (2021). A comparison of ten polygenic score methods for psychiatric disorders applied across multiple cohorts. Biol Psychiatry.

[32] Vandenbroucke J.P., von Elm E., Altman D.G., Gøtzsche P.C., Mulrow C.D., Pocock S.J. (2007). Strengthening the Reporting of Observational Studies in Epidemiology (STROBE): Explanation and elaboration. Epidemiology.

[33] Magnus P., Birke C., Vejrup K., Haugan A., Alsaker E., Daltveit A.K. (2016). Cohort profile update: The Norwegian Mother and Child Cohort Study (MoBa). Int J Epidemiol.

[34] Paltiel L., Anita H., Skjerden T., Harbak K., Bækken S., Nina Kristin S.N. (2014). The biobank of the Norwegian Mother and Child Cohort Study - Present status. Nor Epidemiol.

[35] Bakken I.J., Ariansen A.M.S., Knudsen G.P., Johansen K.I., Vollset S.E. (2020). The Norwegian Patient Registry and the Norwegian Registry for Primary Health Care: Research potential of two nationwide health-care registries. Scand J Public Health.

[36] Corfield E.C., Frei O., Shadrin A.A., Rahman Z., Lin A., Athanasiu L. (2022). The Norwegian Mother, Father, and Child cohort study (MoBa) genotyping data resource: MoBaPsychGen pipeline v.I. bioRxiv.

[37] Hannigan L.J., Corfield E.C., Askelund A.D., Hegemann L., Jensen P., Pettersen J.H. (2023). phenotools: An R package to facilitate efficient and reproducible use of phenotypic data from MoBa and linked registry sources in the TSD environment. Psychgen..

[38] Watson H.J., Diemer E.W., Zerwas S., Gustavson K., Knudsen G.P., Torgersen L. (2019). Prenatal and perinatal risk factors for eating disorders in women: A population cohort study. Int J Eat Disord.

[39] Watson H.J., Torgersen L., Zerwas S., Reichborn-Kjennerud T., Knoph C., Stoltenberg C. (2014). Eating disorders, pregnancy, and the postpartum period: findings from the Norwegian Mother and Child Cohort Study (MoBa). Nor Epidemiol.

[40] Watson H.J., Zerwas S., Torgersen L., Gustavson K., Diemer E.W., Knudsen G.P. (2017). Maternal eating disorders and perinatal outcomes: A three-generation study in the Norwegian Mother and Child Cohort Study. J Abnorm Psychol.

[41] Swahnberg I.M.K., Wijma B. (2003). The NorVold Abuse Questionnaire (NorAQ): Validation of new measures of emotional, physical, and sexual abuse, and abuse in the health care system among women. Eur J Public Health.

[42] Privé F., Arbel J., Vilhjálmsson B.J. (2021). LDpred2: Better, faster, stronger. Bioinformatics.

[43] Allegrini A.G. (2024). LDpred2. GitHub.. https://github.com/AndreAllegrini/LDpred2.

[44] Privé F. (2022). Polygenic scores and inference using LDpred2.. https://privefl.github.io/bigsnpr/articles/LDpred2.html.

[45] Afifi T.O., MacMillan H.L., Boyle M., Taillieu T., Cheung K., Sareen J. (2014). Child abuse and mental disorders in Canada. CMAJ.

[46] Knol M.J., van der Tweel I., Grobbee D.E., Numans M.E., Geerlings M.I. (2007). Estimating interaction on an additive scale between continuous determinants in a logistic regression model. Int J Epidemiol.

[47] Benjamini Y., Yekutieli D. (2001). The control of the false discovery rate in multiple testing under dependency. Ann Statist.

[48] R Core Team (2023).

[49] Udo T., Grilo C.M. (2018). Prevalence and correlates of DSM-5-defined eating disorders in a nationally representative sample of U.S. adults. Biol Psychiatry.

[50] Larsen J.T., Yilmaz Z., Bulik C.M., Albiñana C., Vilhjálmsson B.J., Mortensen P.B., Petersen L.V. (2024). Diagnosed eating disorders in Danish registers - Incidence, prevalence, mortality, and polygenic risk. Psychiatry Res.

[51] VanderWeele T.J., Vansteelandt S. (2014). Invited commentary: Some advantages of the relative excess risk due to interaction (RERI)--Towards better estimators of additive interaction. Am J Epidemiol.

[52] VanderWeele T.J., Knol M.J. (2014). A tutorial on interaction. Epidemiol Methods.

[53] Chuong M., Adams M.J., Kwong A.S.F., Haley C.S., Amador C., McIntosh A.M. (2022). Genome-by-trauma exposure interactions in adults with depression in the UK Biobank. JAMA Psychiatry.

[54] Solmi M., Radua J., Stubbs B., Ricca V., Moretti D., Busatta D. (2021). Risk factors for eating disorders: An umbrella review of published meta-analyses. Braz J Psychiatry.

[55] Stice E., Gau J.M., Rohde P., Shaw H. (2017). Risk factors that predict future onset of each DSM–5 eating disorder: Predictive specificity in high-risk adolescent females. J Abnorm Psychol.

[56] Watson H.J., Palmos A.B., Hunjan A., Baker J.H., Yilmaz Z., Davies H.L. (2021). Genetics of eating disorders in the genome-wide era. Psychol Med.

[57] Baldwin J.R., Reuben A., Newbury J.B., Danese A. (2019). Agreement between prospective and retrospective measures of childhood maltreatment: A systematic review and meta-analysis. JAMA Psychiatry.

[58] Biele G., Gustavson K., Czajkowski N.O., Nilsen R.M., Reichborn-Kjennerud T., Magnus P.M. (2019). Bias from self selection and loss to follow-up in prospective cohort studies. Eur J Epidemiol.

[59] Nilsen R.M., Vollset S.E., Gjessing H.K., Skjærven R., Melve K.K., Schreuder P. (2009). Self-selection and bias in a large prospective pregnancy cohort in Norway. Paediatr Perinat Epidemiol.

[60] Vejrup K., Magnus P., Magnus M. (2022). Lost to follow-up in the Norwegian mother, father and child cohort study. Paediatr Perinat Epidemiol.

[61] Martin J., Taylor M.J., Lichtenstein P. (2018). Assessing the evidence for shared genetic risks across psychiatric disorders and traits. Psychol Med.

