# Supplementary material for: Investigating Relationships Between Genetic Risk, Childhood Maltreatment, and Eating Disorders in Women
Source: Biol Psychiatry Glob Open Sci. 2026 May 25;6(5):100761. doi: 10.1016/j.bpsgos.2026.100761 (PMC13380472; doi:10.1016/j.bpsgos.2026.100761)
Supplement: Supplemental Methods, Figure S1 and Tables S1–S12 [file mmc1.pdf]

SUPPLEMENTARY INFORMATION

Investigating Relationships Between Genetic Risk, Childhood Maltreatment and Eating Disorders in Women

Bjørndal *et al.*

Table of Contents

Overview of deviations from pre-registration.
Figure S1. Study Flow Chart.
Table S1. Preregistration Deviations Table.
Table S2. Sociodemographic Characteristics in Sample at Week 15 of Pregnancy in MoBa.
Table S3. Eating Disorder Outcomes.
Table S4. Model Fitting Strategy.
Table S5. Associations Between PGS Quartiles and Eating Disorder Risk.
Table S6. Interaction Between Childhood Maltreatment and Polygenic Scores (PGS-AN) in Predicting Eating Disorder Risk: Multiplicative Scale (×).
Table S7. Interaction Between Childhood Maltreatment and Polygenic Scores (PGS-BEB) in Predicting Eating Disorder Risk: Multiplicative Scale (×).
Table S8. Associations Between Childhood Maltreatment, PGSs, and Eating Disorder Diagnoses (Registry).
Table S9. Exploratory Analyses of Interaction Effects Between Childhood Maltreatment and PGSs in Predicting Eating Disorders: Additive Scale.
Table S10. Associations Between Childhood Maltreatment and Eating Disorders Without Marital Status Covariate.
Table S11. Associations Between Childhood Maltreatment and Eating Disorders Adjusting for Schizophrenia and Depression Diagnoses.
Table S12. Associations Between PGSs and Eating Disorders Adjusting for Schizophrenia and Depression Diagnoses.

Overview of deviations from pre-registration

We deviated from the pre-registration (available on the Open Science Framework: https://doi.org/10.17605/OSF.IO/EZ6SN

We provide an overview of these deviations and corresponding justifications in Table S1, using a template from Willroth & Atherton.[1]

Table S1.

Preregistration Deviations Table.

**Deviations**					
#	Details		Original Wording	Deviation Description	Reader Impact
1	Type	Variables	We pre-registered to also incorporate a PGS for BMI and assess main effects and potential interaction effects with childhood maltreatment.	We chose not to do this to maintain the focus of the paper on genetic liability to eating disorders.	It should not affect the interpretation of the results.
	Reason	Other (Please Explain)			
	Timing	After data access			
2	Type	Variables	We pre-registered to also incorporate a PGS for binge-eating narrow based on the GWAS by Termorshuizen et al. [2]	We opted to only include the PGS for binge-eating broad as this captured the common genetic component of binge-eating with greater statistical power compared with the binge-eating narrow PGS in the study by Termorshuizen et al. [2] This became clear after the preprint of this study was published which was after our pre-registration was uploaded to OSF.	It should not affect the interpretation of the results.
	Reason	New knowledge			
	Timing	Select One			
3	Type	Select One			

	Reason	Select One			
	Timing	Select One			

Unregistered Steps

#	Details		Original Wording	Unregistered Step Description	Reader Impact
1	Type	Analysis	We did not pre-register to examine additive interaction effects.	We chose to do this following a literature review. Recent studies examining interaction effects between PGSs and stressful life events for mental disorders have examined both multiplicative and additive interaction effects.[3] It has been suggested that additive interactions may more effectively capture the biological mechanisms underlying G × E effects than multiplicative interactions.[4]	As these analyses were not pre-registered, they should be interpreted as exploratory.
	Timing	After results known			
2	Type	Analysis	We did not pre-register to assess agreement in reporting of childhood maltreatment for mothers participating twice.	We chose to do this to provide more information on the consistency of reporting of childhood maltreatment in MoBa.	These results provide contextual information.
	Timing	After results known			
3	Type	Analysis	We did not pre-register to examine	We chose to do this to as a	These results provide contextual

	Timing	After results known	associations between PGSs, childhood maltreatment, and eating disorders with the outcome restricted to diagnosis in the registry.	sensitivity analysis in response to peer review feedback.	information.
4	Type	Analysis	We did not pre-register to conduct analyses examining associations between childhood maltreatment and eating disorders excluding marital status as a covariate	We chose to do this to as a sensitivity analysis in response to peer review feedback.	These results provide contextual information.
	Timing	After results known			

Figure S1.

Flow Chart for Selecting MoBa Participants.

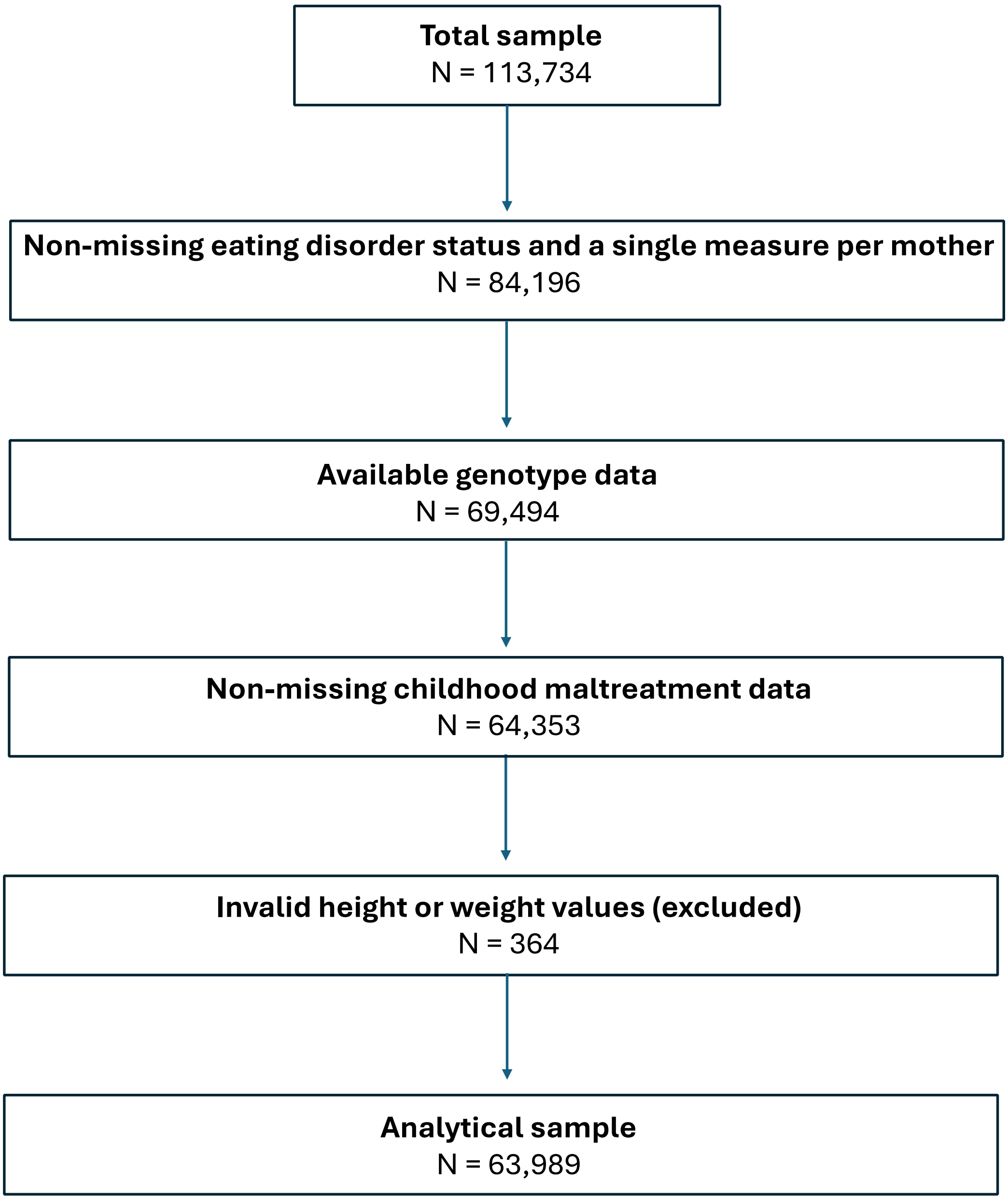

Table S2.

Sociodemographic Characteristics in Sample at Week 15 of Pregnancy in MoBa.

Characteristic	N (%)
Marital status[a]	
Married	46,863 (47.50)
Divorced or separated	272 (0.28)
Cohabiting	47,285 (47.93)
Single	1,908 (1.93)
Widowed and other	980 (0.99)
Education level (completed)[b]	
No higher education	32,704 (33.15)
Higher education	60,129 (60.95)
Self-reported income level[c]	
No income	2,330 (2.4)
< 150,000 NOK	14,650 (14.85)
151,000 – 299,999 NOK	42,505 (43.09)
300,000-499,999 NOK	30,601 (31.02)
> 500,000 NOK	4,351 (4.41)

Note. These numbers were based on all participants with available ED data at one timepoint minimum (N = 98,650). Percentages do not add up to 100 because of missing data for individual variables. [a]Missing data for this variable was N = 1183. [b]Missing data for this variable was N = 5817. [c]Missing data for this variable was N = 4213.

Table S3.

Eating Disorder Outcomes.

Outcome	Description
AN (lifetime risk)	Met criteria for anorexia nervosa (AN) based on survey algorithm at any timepoint (*DSM-5 AN*) and/or had a register-based diagnosis (*ICD-10: F50.0, F50.1*)
BN (lifetime risk)	Met criteria for bulimia nervosa (BN) based on survey algorithm at any timepoint (*DSM-5 BN*) and/or had a register-based diagnosis (*ICD-10: F50.2, F50.3*)
BED (lifetime risk)	Met criteria for binge-eating disorder (BED) based on survey algorithm at any timepoint (*DSM-5 BED*)
PD (lifetime risk)	Met criteria for purging disorder (PD) based on survey algorithm at any timepoint (*DSM-5 OSFED-P*)
BESP (lifetime risk)	Met criteria for BN and/or BED based on survey algorithm at any timepoint (*DSM-5 BN, BED*) and/or received a register-based diagnosis (*ICD-10: F50.2, F50.3*)
No ED (lifetime)	No ED based on survey algorithm at any timepoint (*DSM-5 AN, BN, BED, PD*) and no register-based diagnosis (ICD-10: F50.0, F50.1, F50.2, F50.3, F50.8, F50.9. ICPC-2: P11, T04, T05, P86).

Notes. ICD-10: F50.0 (Anorexia nervosa), F50.1 (Atypical anorexia nervosa), F50.2 (Bulimia nervosa), F50.3 (Atypical bulimia nervosa), F50.8 (Other eating disorders), F50.9 (Eating disorder not otherwise specified). ICPC-2: P11 (Eating disorder in children), T04 (Feeding problem in infant/child), T05 (Feeding problem in adult), P86 (Anorexia nervosa/bulimia). BESP: binge eating spectrum. OSFED-P: other specified feeding or eating disorders–purging disorder.

Table S4.

Model Fitting Strategy.

Model	Fixed effects	Variables
0. Base	CM	Model 0.1: Degradation + Covariates<br>Model 0.2: Threats + Covariates<br>Model 0.3: Physical abuse + Covariates<br>Model 0.4: Sexual abuse + Covariates
1.a Univariable	Single PGS	Model 1.a.1: PGS-AN + Covariates<br>Model 1.a.2: PGS-BEB + Covariates
1.b Multivariable	Multiple PGSs	Model 1.b.1: PGS-AN + PGS-BEB + Covariates
2. Interaction	CM, PGS, CM*PGS	Model 2.1: Degradation + PGS-AN + Covariates<br>Model 2.2: Threats + PGS-AN + Covariates<br>Model 2.3: Physical abuse + PGS-AN + Covariates<br>Model 2.4: Sexual abuse + PGS-AN + Covariates<br>Model 2.5: Degradation + PGS-BEB + Covariates<br>Model 2.6: Threats + PGS-BEB + Covariates<br>Model 2.7: Physical abuse + PGS-BEB + Covariates<br>Model 2.8: Sexual abuse + PGS-BEB + Covariates
3. Quartile	PGS quartile	Model 3.1: PGS-AN (quartiles) + Covariates<br>Model 3.2: PGS-BEB (quartiles) + Covariates

Note. Each model was run for each eating disorder outcome (i.e., AN, BN, BED, PD, BESP). AN: Anorexia nervosa. BEB: binge eating broadly-defined. CM: Childhood maltreatment. PGS: Polygenic score Covariates in each model were: marital status, household income, education level.

Table S5.

Associations Between PGS Quartiles and Eating Disorder Risk.

	**AN**		**BN**		**BED**		**PD**		**BESP**	
**Predictor**	OR [95% CI]	p	OR [95% CI]	p	OR [95% CI]	p	OR [95% CI]	p	OR [95% CI]	p
PGS-AN, Q2	1.24 [1.04, 1.47]	.108	1.11 [0.98, 1.25]	.486	1.08 [1.00, 1.16]	.311	1.32 [0.96, 1.82]	.448	1.09 [1.02, 1.17]	.078
PGS-AN, Q3	1.37 [1.15, 1.62]	.003	1.32 [1.17, 1.48]	<.001	1.06 [0.98, 1.14]	.542	1.29 [0.94, 1.78]	.531	1.11 [1.03, 1.19]	.024
PGS-AN, Q4	1.94 [1.66, 2.29]	<.001	1.40 [1.25, 1.58]	<.001	1.06 [0.99, 1.15]	.531	1.77 [1.31, 2.40]	.002	1.15 [1.07, 1.23]	.001
PGS-BEB, Q2	1.07 [0.91, 1.27]	>.99	1.22 [1.07, 1.39]	.016	1.13 [1.05, 1.22]	.014	1.20 [0.87, 1.65]	>.99	1.15 [1.07, 1.24]	.001
PGS-BEB, Q3	1.20 [1.02, 1.41]	.170	1.41 [1.24, 1.59]	<.001	1.20 [1.11, 1.29]	<.001	1.30 [0.95, 1.79]	.486	1.24 [1.16, 1.33]	<.001
PGS-BEB, Q4	1.43 [1.22, 1.67]	<.001	1.80 [1.60, 2.03]	<.001	1.31 [1.21, 1.41]	<.001	1.66 [1.23, 2.25]	.007	1.41 [1.32, 1.52]	<.001

Note. PGS-AN: Polygenic score for anorexia nervosa. PGS-BEB: Polygenic score for binge-eating broad. p-values were adjusted for multiple testing (number of tests: 30).

Table S6.

Interaction Between Childhood Maltreatment and Polygenic Scores (PGS-AN) in Predicting Eating Disorder Risk: Multiplicative Scale (×).

	AN		BN		BED		PD		BESP	
**Predictor**	OR [95% CI]	p	OR [95% CI]	p	OR [95% CI]	p	OR [95% CI]	p	OR [95% CI]	p
Degradation	1.99 [1.71, 2.30]	<.001	2.19 [1.98, 2.43]	<.001	1.83 [1.70, 1.96]	<.001	1.71 [1.29, 2.24]	<.001	1.86 [1.75, 1.99]	<.001
PGS-AN	1.32 [1.24, 1.40]	<.001	1.14 [1.09, 1.20]	<.001	1.01 [0.98, 1.04]	.72	1.24 [1.10, 1.39]	<.001	1.04 [1.01, 1.07]	<.05
Degradation × PGS-AN	0.98 [0.85, 1.13]	>.99	1.03 [0.93, 1.14]	>.99	1.05 [0.98, 1.13]	>.99	1.00 [0.76, 1.31]	>.99	1.04 [0.98, 1.11]	>.99
Threatened	2.27 [1.82, 2.80]	<.001	2.21 [1.89, 2.58]	<.001	1.84 [1.64, 2.05]	<.001	1.67 [1.05, 2.50]	<.05	1.92 [1.74, 2.12]	<.001
PGS-AN	1.34 [1.26, 1.42]	<.001	1.16 [1.11, 1.21]	<.001	1.01 [0.99, 1.04]	.30	1.23 [1.10, 1.37]	<.001	1.05 [1.03, 1.08]	<.001
Threatened × PGS-AN	0.79 [0.64, 0.98]	>.99	0.89 [0.76, 1.04]	>.99	1.02 [0.91, 1.14]	>.99	1.04 [0.70, 1.56]	>.99	0.98 [0.89, 1.09]	>.99
Physical abuse	2.36 [1.94, 2.85]	<.001	2.22 [1.92, 2.56]	<.001	1.97 [1.78, 2.17]	<.001	2.43 [1.71, 3.37]	<.001	2.01 [1.84, 2.20]	<.001
PGS-AN	1.33 [1.25, 1.41]	<.001	1.15 [1.10, 1.20]	<.001	1.02 [0.99, 1.05]	.23	1.22 [1.09, 1.37]	<.001	1.05 [1.03, 1.08]	<.001
Physical abuse × PGS-AN	0.84 [0.70, 1.02]	>.99	0.98 [0.85, 1.13]	>.99	0.97 [0.88, 1.07]	>.99	1.01 [0.72, 1.40]	>.99	0.97 [0.89, 1.06]	>.99
Sexual abuse	2.71 [2.29, 3.20]	<.001	2.22 [1.94, 2.52]	<.001	1.88 [1.72, 2.06]	<.001	3.35 [2.51, 4.40]	<.001	1.96 [1.81, 2.13]	<.001
PGS-AN	1.34 [1.26, 1.42]	<.001	1.14 [1.09, 1.19]	<.001	1.01 [0.98, 1.04]	.43	1.27 [1.13, 1.43]	<.001	1.05 [1.02, 1.07]	<.001
Sexual abuse × PGS-AN	0.87 [0.74, 1.03]	>.99	1.04 [0.92, 1.19]	>.99	1.02 [0.93, 1.11]	>.99	0.81 [0.61, 1.06]	>.99	1.02 [0.94, 1.11]	>.99

Note. These models adjusted for all covariates (i.e., marital status, education level, household income). AN: Anorexia nervosa. BN: Bulimia nervosa. BED: Binge-eating disorder. PD: Purging disorder. BESP: Binge-eating spectrum disorder. OR: odds ratio. PGS: Polygenic score. BEB: broadly defined binge-eating. The p-values for the interaction effects were adjusted for multiple testing (number of tests: 40).

Table S7.

Interaction Between Childhood Maltreatment and Polygenic Scores (PGS-BEB) in Predicting Eating Disorder Risk: Multiplicative Scale (×).

	**AN**		**BN**		**BED**		**PD**		**BESP**	
**Predictor**	OR [95% CI]	p	OR [95% CI]	p	OR [95% CI]	p	OR [95% CI]	p	OR [95% CI]	p
Degradation	2.01 [1.74, 2.31]	<.001	2.18 [1.96, 2.42]	<.001	1.82 [1.69, 1.95]	<.001	1.76 [1.33, 2.30]	<.001	1.86 [1.74, 1.98]	<.001
PGS-BEB	1.16 [1.09, 1.24]	<.001	1.24 [1.18, 1.30]	<.001	1.09 [1.06, 1.12]	<.001	1.30 [1.15, 1.46]	<.001	1.13 [1.10, 1.16]	<.001
Degradation × PGS-BEB	0.91 [0.79, 1.05]	>.99	1.02 [0.92, 1.13]	>.99	1.02 [0.95, 1.10]	>.99	0.83 [0.63, 1.08]	>.99	1.01 [0.94, 1.07]	>.99
Threatened	2.22 [1.78, 2.73]	<.001	2.21 [1.88, 2.58]	<.001	1.82 [1.63, 2.04]	<.001	1.64 [1.03, 2.48]	<.05	1.92 [1.73, 2.12]	<.001
PGS-BEB	1.17 [1.10, 1.24]	<.001	1.25 [1.20, 1.31]	<.001	1.09 [1.06, 1.13]	<.001	1.25 [1.12, 1.39]	<.001	1.13 [1.11, 1.16]	<.001
Threatened × PGS-BEB	0.73 [0.59, 0.90]	.69	0.93 [0.80, 1.09]	>.99	1.04 [0.93, 1.16]	>.99	1.09 [0.72, 1.66]	>.99	0.98 [0.89, 1.09]	>.99
Physical abuse	2.32 [1.91, 2.80]	<.001	2.19 [1.89, 2.53]	<.001	1.96 [1.78, 2.16]	<.001	2.44 [1.70, 3.39]	<.001	2.00 [1.83, 2.19]	<.001
PGS-BEB	1.16 [1.10, 1.24]	<.001	1.24 [1.19, 1.30]	<.001	1.10 [1.07, 1.13]	<.001	1.26 [1.13, 1.41]	<.001	1.13 [1.10, 1.16]	<.001
Physical abuse × PGS-BEB	0.80 [0.67, 0.97]	>.99	1.02 [0.89, 1.18]	>.99	0.99 [0.90, 1.09]	>.99	0.99 [0.72, 1.38]	>.99	0.99 [0.90, 1.08]	>.99
Sexual abuse	2.69 [2.28, 3.17]	<.001	2.21 [1.94, 2.52]	<.001	1.88 [1.72, 2.06]	<.001	3.41 [2.56, 4.48]	<.001	1.97 [1.81, 2.14]	<.001
PGS-BEB	1.16 [1.09, 1.24]	<.001	1.24 [1.19, 1.30]	<.001	1.10 [1.07, 1.13]	<.001	1.33 [1.18, 1.50]	<.001	1.14 [1.11, 1.17]	<.001
Sexual abuse × PGS-BEB	0.85 [0.72, 1.00]	>.99	0.99 [0.87, 1.12]	>.99	0.93 [0.85, 1.02]	>.99	0.70 [0.53, 0.91]	.80	0.94 [0.86, 1.01]	>.99

Note. These models adjusted for all covariates (i.e., marital status, education level, household income). AN: Anorexia nervosa. BN: Bulimia nervosa. BED: Binge-eating disorder. PD: Purging disorder. BESP: Binge-eating spectrum disorder. OR: odds ratio. PGS: Polygenic score. BEB: broadly defined binge-eating. The p-values for the interaction effects were adjusted for multiple testing (number of tests: 40).

Table S8.

Associations Between Childhood Maltreatment, PGSs, and Eating Disorder Diagnoses (Registry).

	Any eating disorder diagnosis (registry)	
**Predictor**	OR [95% CI]	p
Degradation	2.19 [1.82, 2.62]	<.001
Threatened	2.44 [1.88, 3.11]	<.001
Physical abuse	2.59 [2.06, 3.23]	<.001
Sexual abuse	2.15 [1.72, 2.67]	<.001
PGS-AN	1.22 [1.13, 1.31]	<.001
PGS-BEB	1.34 [1.24, 1.45]	<.001

Table S9.

Exploratory Analyses of Interaction Effects Between Childhood Maltreatment and PGSs in Predicting Eating Disorders: Additive Scale.

Predictor	AN<br>RERI<br>[95% CI]a	BN<br>RERI<br>[95% CI]a	BED<br>RERI<br>[95% CI]a	PD<br>RERI<br>[95% CI]a	BESP<br>RERI<br>[95% CI]a
Degradation, PGS-AN	0.25<br>[-0.08, 0.61]	0.25<br>[0.04, 0.51]	0.10<br>[-0.03, 0.22]	0.16<br>[-0.32, 0.77]	0.12<br>[0.00, 0.25]
Threatened, PGS-AN	-0.20<br>[-0.64, 0.31]	-0.09<br>[-0.39, 0.25]	0.05<br>[-0.14, 0.24]	0.24<br>[-0.58, 1.16]	0.02<br>[-0.16, 0.19]
Physical abuse, PGS-AN	-0.05<br>[-0.52, 0.49]	0.13<br>[-0.17, 0.49]	-0.04<br>[-0.22, 0.17]	0.34<br>[-0.49, 1.21]	-0.00b<br>[-0.17, 0.19]
Sexual abuse, PGS-AN	0.12<br>[-0.35, 0.65]	0.28<br>[-0.01, 0.64]	0.04<br>[-0.11, 0.22]	-0.19<br>[-1.03, 0.96]	0.09<br>[-0.06, 0.27]
Degradation, PGS-BEB	-0.04<br>[-0.31, 0.25]	0.33<br>[0.11, 0.57]	0.12<br>[-0.01, 0.26]	-0.17<br>[-0.60, 0.37]	0.13<br>[0.00, 0.25]
Threatened, PGS-BEB	-0.49<br>[-0.80, -0.03]	0.12<br>[-0.24, 0.55]	0.15<br>[-0.07, 0.38]	0.35<br>[-0.56, 1.36]	0.09<br>[-0.11, 0.30]
Physical abuse, PGS-BEB	-0.31<br>[-0.66, 0.11]	0.35<br>[-0.01, 0.73]	0.07<br>[-0.13, 0.28]	0.35<br>[-0.59, 1.48]	0.11<br>[-0.08, 0.32]
Sexual abuse, PGS-BEB	-0.19<br>[-0.57, 0.24]	0.26<br>[-0.05, 0.59]	-0.05<br>[-0.20, 0.11]	-0.58<br>[-1.39, 0.45]	-0.01<br>[-0.16, 0.16]

Note. [a]Bootstrapped CI (based on 1,000 bootstrapped samples). [b]RERI = -0.002. AN: Anorexia nervosa. BN: Bulimia nervosa. BED: Binge-eating disorder. PD: Purging disorder. BESP: Binge-eating spectrum disorders. RERI: Relative Excess due to Interaction. PGS: Polygenic score. BEB: binge-eating broad. These tests were not pre-registered. RERI estimates the relative excess risk due to interaction. RERI > 0 indicates a positive additive interaction (combined effect exceeds the sum of individual effects), RERI < 0 indicates a negative additive interaction (combined effect is less than the sum), and RERI = 0 indicates no additive interaction.

Table S10.

Associations Between Childhood Maltreatment and Eating Disorders Without Marital Status Covariate.

Predictor	AN<br>OR<br>[95% CI]	BN<br>OR<br>[95% CI]	BED<br>OR<br>[95% CI]	PD<br>OR<br>[95% CI]	BESP<br>OR<br>[95% CI]
Degradation	2.01<br>[1.74, 2.31]	2.23<br>[2.01, 2.46]	1.84<br>[1.71, 1.98]	1.74<br>[1.32, 2.27]	1.88<br>[1.76, 2.01]
Threatened	2.21<br>[1.78, 2.72]	2.23<br>[1.90, 2.60]	1.87<br>[1.67, 2.08]	1.74[a]<br>[1.12, 2.58]	1.95<br>[1.76, 2.15]
Physical abuse	2.30<br>[1.89, 2.77]	2.24<br>[1.94, 2.58]	1.98<br>[1.79, 2.18]	2.50<br>[1.77, 3.44]	2.02<br>[1.85, 2.21]
Sexual abuse	2.70<br>[2.29, 3.17]	2.26<br>[1.99, 2.57]	1.89<br>[1.72, 2.06]	3.29<br>[2.47, 4.32]	1.98<br>[1.82, 2.14]

Note. AN: anorexia nervosa. BN: bulimia nervosa. BED: binge-eating disorder. PD: purging disorder: BESP: binge-eating spectrum disorders (i.e., BN and/or BED). OR: odds ratio. All p-values < 0.001 except for [a].009.

Table S11.

Associations Between Childhood Maltreatment and Eating Disorders Adjusting for Schizophrenia and Depression Diagnoses.

Predictor	AN<br>OR<br>[95% CI]	BN<br>OR<br>[95% CI]	BED<br>OR<br>[95% CI]	PD[a]<br>OR<br>[95% CI]	BESP<br>OR<br>[95% CI]
Degradation	1.92<br>[1.66, 2.22]	2.12<br>[1.91, 2.35]	1.78<br>[1.66, 1.91]	1.67<br>[1.26, 2.18]	1.81<br>[1.70, 1.93]
Threatened	2.12<br>[1.70, 2.61]	2.07<br>[1.76, 2.42]	1.78<br>[1.59, 1.98]	1.64[b]<br>[1.06, 2.44]	1.85<br>[1.67, 2.04]
Physical abuse	2.18<br>[1.79, 2.63]	2.09<br>[1.81, 2.41]	1.90<br>[1.72, 2.09]	2.37<br>[1.68, 3.28]	1.93<br>[1.76, 2.11]
Sexual abuse	2.55<br>[2.15, 3.00]	2.12<br>[1.86, 2.41]	1.82<br>[1.66, 2.09]	3.18<br>[2.38, 4.18]	1.89<br>[1.74, 2.05]

Note. AN: anorexia nervosa. BN: bulimia nervosa. BED: binge-eating disorder. PD: purging disorder: BESP: binge-eating spectrum disorders (i.e., BN and/or BED). These models adjusted for (in addition to marital status, education level, and household income) having any of the following schizophrenia diagnoses (F20.0, F20.1, F20.2, F20.3, F20.5, F20.8, F20.9) and depression diagnoses (F32.0, F32.1, F32.2, F32.3, F32.8, F32.9) recorded in the Norwegian Patient Registry (lifetime). OR: odds ratio. [a]Models with PD as the outcome did not adjust for schizophrenia diagnoses as this led to instability in model estimation. All p-values < 0.001 except for [b].020.

Table S12.

Associations Between PGSs and Eating Disorders Adjusting for Schizophrenia and Depression Diagnoses.

	AN		BN		BED		PD[a]		BESP	
Predictor	OR [95% CI]	p	OR [95% CI]	p	OR [95% CI]	p	OR [95% CI]	p	OR [95% CI]	p
PGS-AN	1.31 [1.23, 1.38]	<.001	1.15 [1.10, 1.19]	<.001	1.01 [0.99, 1.04]	.351	1.23 [1.11, 1.37]	<.001	1.05 [1.02, 1.07]	<.001
PGS-BEB	1.14 [1.07, 1.20]	<.001	1.24 [1.19, 1.29]	<.001	1.09 [1.06, 1.12]	<.001	1.25 [1.12, 1.39]	<.001	1.13 [1.10, 1.16]	<.001

Note. AN: anorexia nervosa. BN: bulimia nervosa. BED: binge-eating disorder. PD: purging disorder: BESP: binge-eating spectrum disorders (i.e., BN and/or BED). PGS: Polygenic score. BEB: broadly defined binge-eating. These models adjusted for (in addition to marital status, education level, and household income) having any of the following schizophrenia diagnoses (F20.0, F20.1, F20.2, F20.3, F20.5, F20.8, F20.9) and depression diagnoses (F32.0, F32.1, F32.2, F32.3, F32.8, F32.9) recorded in the Norwegian Patient Registry (lifetime). OR: odds ratio. [a]Models with PD as the outcome did not adjust for schizophrenia diagnoses as this led to instability in model estimation.

References

1. Willroth EC, Atherton OE. Best Laid Plans: A Guide to Reporting Preregistration Deviations. *Adv Methods Pract Psychol Sci*. 2024;7(1):25152459231213802. doi:10.1177/25152459231213802

2. Termorshuizen JD, Davies HL, Lee SH, et al. Genome-wide association studies of binge-eating behaviour and anorexia nervosa yield insights into the unique and shared biology of eating disorder phenotypes. Published online February 4, 2025:2025.01.31.25321397. doi:10.1101/2025.01.31.25321397

3. Musliner KL, Andersen KK, Agerbo E, et al. Polygenic liability, stressful life events and risk for secondary-treated depression in early life: a nationwide register-based case-cohort study. *Psychol Med*. 2023;53(1):217-226. doi:10.1017/S0033291721001410

4. Mullins N, Power RA, Fisher HL, et al. Polygenic interactions with environmental adversity in the aetiology of major depressive disorder. *Psychol Med*. 2016;46(4):759-770. doi:10.1017/S0033291715002172
